# Dental service utilization among hospitalized patients with psychiatric and/or substance use disorders: a Norwegian cross-sectional study

**DOI:** 10.3389/froh.2026.1904583

**Published:** 2026-08-10

**Authors:** Kristina G. Kantola, Rolf Wynn, Hege Nermo, Jan-Are Kolset Johnsen, Elin Hadler-Olsen

**Affiliations:** 1 The Public Dental Health Service Competence Center of Northern Norway, Tromsø, Norway; 2 Department of Clinical Medicine, Faculty of Health Sciences, UiT The Arctic University of Norway, Tromsø, Norway; 3 Department of Clinical Medicine, University Miguel Hernandez, Alicante, Spain; 4 Department of Clinical Dentistry, Faculty of Health Sciences, UiT The Arctic University of Norway, Tromsø, Norway; 5 Department of Medical Biology, Faculty of Health Sciences, UiT The Arctic University of Norway, Tromsø, Norway

**Keywords:** dental care, dental health services, health services accessibility, mental disorders, oral health, substance-related disorders

## Abstract

**Introduction:**

Individuals with psychiatric and/or substance use disorders often have poor oral health, yet utilization of dental services remains low. This study examined dental attendance, barriers to care, and factors associated with regular dental care among inpatients in Norway.

**Methods:**

This cross-sectional study included 138 adults hospitalized in specialist mental health and substance use services at the University Hospital of North Norway (median age 35.5 years, range 19–70, 60% men, 40% women). Participants completed a structured questionnaire covering various health domains and underwent clinical and radiographic oral examinations. Associations with irregular dental care, defined as attending dental services regularly less than every second year, were examined using block-wise logistic regression guided by Andersen's Behavioral Model of Health Service Use to evaluate the relative contribution of predisposing, enabling, behavioral, and need-related factors. The study was approved by the Regional Committee for Medical and Health Research Ethics, and all participants provided written informed consent.

**Results:**

Regular dental care was reported by 28.7%, while 49.3% sought care for acute problems only. Financial constraints and dental fear were the main barriers to regular dental care. Negative experiences with dental care were frequently reported. In multivariable regression, lack of or unknown entitlement to free dental care was strongly associated with irregular care (OR = 3.15, 95% CI, 1.30–7.64). Lower toothbrushing frequency (OR = 2.53, 95% CI, 1.06–6.02) and low educational attainment (OR = 4.13, 95% CI, 1.03–16.62) were also associated with irregular care. After adjustment, neither self-reported dental health nor clinically assessed dental health was associated with regular dental care.

**Conclusion:**

Regular dental care was low despite poor oral health and was more strongly associated with entitlement to free dental care, toothbrushing frequency, and educational attainment than with clinical indicators of treatment need. Improving dental service utilization in this population may require not only financial coverage, but also clearer pathways into care and patient-centered approaches that promote safety, trust, and sustained engagement with dental services.

## Introduction

Individuals with severe psychiatric disorders and/or substance use disorders often experience poor oral health compared with the general population, with dental caries, tooth loss (1–4), orofacial pain (5, 6) and xerostomia (4) being common. These conditions may contribute to the substantial oral health-related quality of life impairments observed in this population, affecting essential daily functions such as eating, speaking, sleeping, and social interaction (7–9).

Unlike many other healthcare services, regular preventive dental care is recommended even in the absence of symptoms, due to the high incidence and progressive nature of oral diseases. In Norway, health authorities generally recommend dental examinations at intervals not exceeding two years (10). Despite considerable oral health needs, individuals with psychiatric and/or substance use disorders tend to use dental services less frequently (11) and less regularly than the general population (12). This mismatch suggests substantial unmet treatment needs and barriers to accessing care.

In Norway, access to publicly funded dental care for adults is limited and based on specific entitlement schemes. For individuals with psychiatric disorders and/or substance use disorders, eligibility for free dental care may depend on factors such as diagnosis, treatment setting, duration of institutional stay, or municipal health services involvement. As a result, access to care may be difficult to navigate for individuals with substantial health and social challenges.

Previous research suggests that dental service utilization is influenced by a complex interplay of factors (11–16). In population-based studies, dental attendance has been proposed as a key pathway linking socio-economic status to oral health outcomes, partly mediated through perceived barriers to care (13). Andersen's Behavioral Model of Health Service Use (14, 15) provides a well-established conceptual framework for studying health service utilization and has been widely applied in research of dental service utilization (17, 18) The model distinguishes between predisposing, enabling, behavioral, and need-related factors. Enabling factors are considered particularly important, as they directly influence an individual's ability to access care (15). Thus, variables in the present study were grouped according to these conceptual domains to guide the block-wise regression analysis.

Psychosocial and experiential factors also appear important. Dental anxiety (19–21), previous negative experiences with dental care (19, 22, 23), and discomfort in interactions with healthcare providers (11) have all been identified as barriers to care.

Furthermore, several factors related to psychiatric illness, substance use, and their management may contribute to reduced dental service utilization. These include loss of activity and initiative among substance users (24), negative symptoms among patients with schizophrenia (25), coercive treatment contexts, including involuntary admission or treatment, which may affect trust in healthcare providers (26), and shame within healthcare systems, which may further reduce help-seeking and engagement with care (11). In addition, individuals in this population often experience long-term illness, with many affected over substantial parts of their lives. This, in combination with financial constraints and other life challenges, may further limit their ability to seek and maintain regular dental attendance (16).

Although several studies have explored barriers to dental care among individuals with psychiatric and/or substance use disorders, important gaps remain regarding psychiatric inpatients, a subgroup characterized by more severe and complex clinical presentations. Admission to inpatient care typically reflects acute or severe illness, often associated with substantial functional impairment and instability over time. These factors may further challenge the ability to initiate and maintain regular dental care, distinguishing this group from individuals with milder conditions living in the community.

Furthermore, individuals in inpatient settings are often underrepresented in research, contributing to limited knowledge about how they use dental services, which factors influence their utilization, and how they experience access to care. A more comprehensive understanding that integrates both patterns of service use and associated determinants is therefore needed.

The aim of this study was to examine dental service utilization among inpatients with psychiatric and/or substance use disorders. Specifically, the study aimed to: (1) describe patterns of dental service utilization, including frequency of attendance and type of service provider; (2) assess patient-reported barriers to dental care; (3) explore patients' experiences with dental services; (4) identify factors perceived as important for receiving care; and (5) examine factors associated with regular dental care using multivariable regression analyses based on Andersen's model.

## Material and methods

This study is reported in accordance with the Strengthening the Reporting of Observational Studies in Epidemiology (STROBE) statement (27).

### Study design and recruitment

This cross-sectional study was conducted among adult inpatients receiving care within the Division of Mental Health and Substance Use at the University Hospital of North Norway. Data collection took place from October 2021 to July 2023. Participants completed a structured interview administrated by a trained assistant and underwent both clinical and radiographic oral health examinations. Further methodological details have been described previously (3).

Eligible participants were adults (≥18 years) admitted to selected units, including acute psychiatric wards, security units, wards for co-occurring substance use and severe mental disorders, and specialized substance use treatment units. Patients younger than 18 years, those admitted to geriatric psychiatric services, or individuals lacking the capacity to provide informed consent were excluded.

Information about the study was disseminated through ward personnel, posters and leaflets in shared areas. For patients who expressed interest, healthcare staff facilitated initial contact with dental personnel. Prior to providing informed consent to participate, participants received further written and oral information about the study. Participants were offered complimentary dental cleaning and a gift card (200 NOK, approximately €17) as compensation.

The study population consisted of patients hospitalized due to severe psychiatric conditions and/or substance use disorders, representing a group with substantial clinical complexity. Only patients deemed clinically stable and capable of participating at the time of recruitment were included.

Participants were recruited using a convenience sampling approach. The final sample comprised all eligible patients who consented to participate during the study period. No formal record was kept of the number of eligible patients approached or those who declined participation.

### Procedures

All clinical oral examinations were performed by a single trained dentist. As dental and medical records were stored in separate systems, the examiner had no access to participants' psychiatric diagnoses, and questionnaire data were not available during the clinical examination. These procedures minimized the risk of observational bias.

The oral examination consisted of a direct clinical examination performed by the examiner, supplemented by four bitewing radiographs. Standardized clinical photographs (Nikon D7000; AF-S Micro Nikkor 105 mm 1:2.8G ED) were obtained as supplementary documentation. Structured interviews were carried out by two trained assistants using standardized questionnaires. The interviewers aimed to maintain a neutral approach and provided clarification when needed to ensure consistent understanding of the questions.

### Variables

#### Dental service utilization, experiences, and access to care

***Regular dental care:*** Dental service utilization was assessed with the question: “Do you attend dental services regularly (dentist/dental hygienist)?” Response options were: (1) more than once a year, (2) once a year, (3) every second year, (4) less frequently than every second year, (5) only for acute problems, and (6) never.

To reflect recommended recall intervals and ensure sufficient group sizes for analysis, responses were dichotomized into: (1) regular attendance (at least every second year) and (2) irregular attendance (less than every second year, attendance only for acute problems, or never).

***Pain experience at dentist***: Participants were asked to rate how painful they generally perceive dental visits, on a scale from 0 (no pain) to 10 (worst imaginable pain).

***Adverse dental experiences (Loe-DEQ):*** Adverse dental experiences were assessed using selected items from the Level of Exposure–Dental Experiences Questionnaire (LOE-DEQ) (28), reflecting negative experiences during dental treatment were including, pain, lack of control, negative interactions with dental staff, insufficient information, and physiological reactions (e.g., dizziness or nausea). Each item was scored dichotomously (yes/no), and a sum score was calculated, reflecting the overall level of adverse dental experiences.

***Entitlement to publicly funded dental care:*** Entitlement to free dental treatment within the public dental service (PDS) was assessed with response options: (1) yes, (2) no, or (3) do not know. For analysis, responses were recategorized as: (1) entitled and (2) not entitled/do not know.

***Entitlement to reimbursement for dental care from the Norwegian National Insurance Scheme:*** Participants were asked if they were eligible for partial reimbursement of dental care costs through the Norwegian National Insurance Scheme, administered by the Norwegian Health Economics Administration (Helfo), with the response options (1) yes, (2) no, or (3) do not know. For analysis, they were dichotomized as (1) entitled and (2) not entitled/do not know.

***Factors considered important for receiving dental care:*** Participants rated the importance of various factors that could facilitate dental health care use. Items covered affordability of treatment, having a regular dentist, receiving sufficient information, feeling safe and in control during treatment, practical support with appointments, accompaniment to appointments, and access to sedation or general anesthesia. Responses were recorded on a 5-point scale ranging from 0 (not important) to 4 (absolutely essential). For descriptive analyses, responses were categorized as of little importance (0–1), quite important (2), and very important (3–4).

### Sociodemographic and health variables

***Sex and age*** were obtained from the questionnaire. Sex was categorized as female or male, and age was a continuous variable.

***Education:*** Educational attainment was categorized into four levels: (1) primary school, (2) upper secondary school or vocational education, (3) college or university <4 years, and (4) college or university ≥4 years. For statistical analyses, categories (3) and (4) were combined to represent higher education.

***Financial situation:*** Financial situation was assessed with five response options: (1) Very good, (2) Good, (3) Average, (4) Difficult, (5) Very difficult. For statistical analyses, responses were dichotomized into: (1) Good/Average (original options 1–3) and (2) Difficult (original options 4 and 5).

***Smoking:*** Smoking status was assessed with four response options: (1) never, (2) former smoker, (3) occasional smoker, and (4) daily smoker. For statistical analyses, they were dichotomized into (1) Non-smoker (never/former) and (2) Current smoker (occasional/daily).

***Tooth brushing frequency:*** Toothbrushing frequency was assessed with options: (1) less than once a week, (2) a few times a week, (3) once a day, and (4) twice a day or more For analysis, responses were dichotomized into: (1) less than twice daily (original options 1–3) and (2) at least twice daily (original option 4).

***Alcohol consumption:*** Alcohol consumption was assessed using the validated Alcohol Use Disorders Identification Test–Consumption (AUDIT-C) (29–31). The instrument includes items on drinking frequency and typical alcohol intake over the past 12 months, yielding a total score ranging from 0 to 12.

***Substance use:*** Participants reported their use of drugs or addictive medications (excluding alcohol) with the options: (1) never, (2) tried occasionally, (3) once a month or less, (4) 2–4 times a month, (5) 2–3 times a week, (6) 4 times a week or more, and (7) use during relapse periods. Responses were dichotomized into: (1) never (option 1) and (2) current or episodic use (original options 2–7), including use during relapse periods.

***Psychological distress:*** Symptoms of anxiety and depression were assessed using the 10-item Hopkins Symptom Checklist (32). A sum-score for all items was calculated.

***PQ-16 score:*** Symptoms related to psychosis were assessed using the 16-item Prodromal Questionnaire (PQ-16) (33–35). Responses were summed to obtain a total score. While the PQ-16 is developed to screen for prodromal psychotic symptoms in non-hospital settings, the present inpatient sample included individuals with both early symptoms and established psychotic disorders. To account for this, the measure is referred to as a PQ-16 score, reflecting overall symptom burden rather than prodromal risk.

***Xerostomia:*** Participants were asked if they were bothered by dry mouth, with the four options: (1) not at all, (2) a little, (3) quite, and (4) very bothered. For analyses, responses were dichotomized into: (1) no or mild xerostomia (original options 1–2) and (2) moderate to severe xerostomia (original options 3–4).

***Self-reported dental health and general health:*** Participants were asked to rate their dental health and general health, each with the options: (1) very good, (2) good, (3) neither good nor poor, (4) poor, and (5) very poor. For analysis, responses were dichotomized into: (1) good (original options 1–2) and (2) moderate/poor (original options 3–5) for both variables.

***Orofacial pain*:** Orofacial pain and dysfunction were assessed with three questions (36, 37): (1) pain in the temples, face, jaw, or jaw joint once a week or more often; (2) pain when opening the mouth wide or chewing once a week or more often; and (3) jaw locking or catching once a week or more often. All items were answered dichotomously (yes/no). A composite variable was created, where a “yes” to any of the items was coded as pain/discomfort, and “no” to all items was coded as no pain/discomfort.

***Modified Dental Anxiety Scale (MDAS):*** Dental anxiety was assessed using the Modified Dental Anxiety Scale (MDAS) (38). The instrument consists of five items measuring anxiety related to different dental situations, each scored on a five-point Likert scale (1–5), yielding a total score ranging from 5 to 25. For analyses, the total score was dichotomized using an established cutoff for severe dental anxiety (39): (1) low to moderate dental anxiety (<19) and (2) severe dental anxiety (≥19).

### Clinical oral health variables

The 3rd molars were excluded from the analysis. Caries, restorations, and missing teeth were recorded based on findings from the clinical examination and bitewing radiographs, supported by standardized clinical photographs, as previously described (3).

The intra-examiner agreement for caries assessment was high. Intraclass correlation coefficients (ICC) were 0.875 (95% CI, 0.862–0.888) for primary caries and 0.783 (95% CI, 0.759–0.805) for secondary caries. Procedures related to radiographic standardization, examiner calibration, and assessment of intra-examiner reliability have been described in detail in a previous publication (3).

***Decayed teeth (DT):*** Dental caries was graded on a five-point scale according to lesion depth: (1) outer enamel, (2) inner enamel, (3) outer dentin, (4) middle dentin, and (5) inner dentin. Teeth with caries extending into dentin (scores 3–5) were classified as decayed teeth (DT) and summarized as the total number of decayed teeth per participant.

***Missing teeth (MT):*** were recorded irrespective of reason for tooth loss and summarized.

***Filled teeth (FT):*** included all teeth with permanent restorations, including crowns and bridge abutments. Teeth with restorations and concurrent caries extending into dentin were classified as decayed rather than filled.

***Decayed, missing, and filled teeth (DMFT):*** The DMFT index represents the total number of decayed (DT), missing (MT), and filled (FT) teeth per participant representing cumulative caries experience.

### Statistical analysis

IBM SPSS Statistics version 29.0.0.0 (241) was used for all statistical analyses.

Continuous variables were reported as medians with 25th and 75th percentiles due to non-normal distributions and differences across dental attendance groups were assessed using the non-parametric Mann–Whitney U test and Kruskal–Wallis test. Differences in categorical variables across groups were analyzed using cross tabulations and chi-square tests. Analyses were conducted using complete case analysis (listwise deletion), whereby cases with missing data on variables included in each analysis were excluded. Given the low proportion of missing data (<4% for all variables; range 0.7%—3.6%), complete case analysis was considered an appropriate analytical approach.

### Block-wise logistic regression analysis

Univariate analyses were performed as a preparatory step for regression analyses. Categories with small numbers of participants were merged to ensure adequate cell sizes and statistical stability, guided by conceptual similarity and similarity in distributions across outcome groups.

A block-wise logistic regression analysis was conducted to examine factors associated with dental service utilization. Variables considered for the multivariable regression model were selected *a priori* based on Andersen's Behavioral Model of Health Service Use, previous literature on dental service utilization (11, 12, 16, 23, 40, 41), and the aim of maintaining a parsimonious model. Given the modest sample size, the number of predictors included in the model was restricted to avoid overfitting. Consequently, not all variables included in the descriptive analyses were entered into the multivariable model. Variables presented solely to describe participant characteristics, experiences, or perceived barriers were not considered candidates for multivariable modelling. Guided by Andersen's Behavioral Model of Health Service Use, variables were entered sequentially in blocks: Block 1 consisted of predisposing factors (sex, age, educational attainment); Block 2 consisted of enabling factors (entitlement to publicly funded dental treatment, reimbursement eligibility through the Norwegian National Insurance Scheme); Block 3 consisted of behavioral factors (smoking status, toothbrushing frequency and alcohol consumption); and Block 4 consisted of need factors (self-reported dental health and the clinical oral health variable DMFT). DMFT was selected as the clinical oral health variable because it is the most widely used summary measure of cumulative caries experience. At each step, the incremental contribution of the newly added block was evaluated using changes in model fit (*Δχ*^2^). Model performance and explanatory power were assessed using Nagelkerke's R^2^, and model calibration was evaluated using the Hosmer–Lemeshow goodness-of-fit test.

This block-wise approach was used to assess the relative contribution of each Andersen's model domain beyond the preceding blocks. The final model included all variables simultaneously. Results are presented as odds ratios (ORs) with 95% confidence intervals (CI).

Multicollinearity among predictors was assessed using variance inflation factors (VIF) derived from auxiliary linear regression models. All VIF values were below 2, indicating no evidence of problematic collinearity. Sensitivity analyses were performed by replacing DMFT with its individual components (DT, MT, and FT) in the final regression model to assess the robustness of the findings. In addition, bootstrap resampling [1,000 samples using bias-corrected and accelerated (BCa) confidence intervals] was performed for the parsimonious and extended logistic regression models as a sensitivity analysis to assess the stability of the parameter estimates.

The HSCL-10 demonstrated good internal consistency (Cronbach's *α* = 0.88), with all items showing acceptable item–total correlations (>0.30). The PQ-16 also showed good internal consistency (Cronbach's *α* = 0.90), supporting the use of a summed score.

The dental trauma items demonstrated acceptable internal consistency (Cronbach's *α* = 0.74), indicating that the items capture related aspects of adverse dental experiences. The AUDIT-C demonstrated good internal consistency (Cronbach's *α* = 0.89), with strong item–total correlations. Missing values in alcohol-related items resulting from skip patterns among non-drinkers were recoded as zero to reflect absence of alcohol use.

Linearity in the logit was assessed for all continuous variables using the Box–Tidwell procedure, and the assumption was met for all variables. Model diagnostics were assessed using standardized residuals, Cook's distance, and leverage values. No influential observations were identified, and no cases were excluded from the analyses. Overall, the assumptions for logistic regression were considered satisfactory.

## Ethics

The study was approved by the Regional Committee for Medical and Health Research Ethics (approval reference number 240987, dated 16.06.2021) and the Norwegian Centre for Research Data (approval reference number 119768, dated 12.08.2021). Written informed consent was obtained from all participants.

## Results

The study included 138 participants with a median age 35.5 years (range: 19–70), 83 (60.1%) men and 55 (39.9%) women.

### Dental service utilization and barriers to care

Regular dental care was uncommon in the study population, with 28.7% attending at least every second year. Notably, 49.3% sought dental care only for acute problems, and 6.6% reported never attending. About half of the participants received care within the PDS (54.0%), 21.2% used private services and 19.7% reported a combination of both. The remaining 5.1% reported that they hardly ever visited a dentist or dental hygienist.

Among those who had regular dental care less than every second year, financial constraints (34.7%) and dental fear (22.1%) were the most reported reasons for not having more frequent visits. Other reasons included low prioritization of dental care (18.9%) and perceived lack of need (13.7%). No participants reported difficulties obtaining an appointment. The reported barriers were reflected in behavior where 50% of the participants had postponed dental visits due to financial constraints, and 32.8% due to dental fear within the past two years. The prevalence of high dental anxiety (MDAS ≥ 19) was 13.2%.

### Experiences with dental care

Negative experiences with dental care were frequently reported. Painful or frightening dental treatment was experienced by 64%, 50% had felt helpless, and 42% had felt ashamed during dental visits. Additionally, 26.6% had experienced physical discomfort such as dizziness, nausea, or choking, and 30% described encounters with dental personnel as rude, critical, or condescending.

When asked to rate how painful a dental visit tended to be on a scale from 1 to 10, the mean score was 3.7 (SD 3.2), with a median of 3.0 (range: 1.0–5.0).

### Perceived facilitators of access to dental care

When asked about factors facilitating access to dental care (Table 1), affordability was the factor most frequently rated as very important. Feeling in control during treatment, receiving sufficient explanation, and having a regular dentist who felt safe were also commonly perceived as very important. In addition, almost one third considered practical support with scheduling and remembering appointments very important. Approximately 40% considered treatment under sedation or general anesthesia important or very important.

**Table 1:** Factors considered important for receiving dental treatment.

How important are the following factors for you to be able to receive dental treatment?	Very important	Quite important	Of little importance
	*n* (%)	*n* (%)	*n* (%)
That the treatment is free or affordable	91 (66.4)	26 (19.0)	20 (14.6)
That I have a regular dentist I feel safe with	64 (46.7)	50 (36.5)	22 (16.1)
That I feel the dentist has enough time to explain what will happen	65 (47.4)	46 (33.6)	22 (16.1)
That I feel in control during treatment and can stop at any time	69 (50.4)	46 (33.6)	22 (16.1)
That I receive help to schedule and remember dental appointments	43 (31.4)	47 (34.3)	47 (34.3)
That I have someone who can accompany me to dental appointments	14 (10.2)	12 (8.8)	111 (81.0)
That I can receive sedatives or general anesthesia during treatment	28 (20.4)	31 (22.6)	78 (56.9)

Participants' ratings of the importance of factors facilitating receipt of dental treatment. Data are presented as *n* (%).

Table 2 presents participant characteristics by regular dental care (at least every second year vs. less frequently).

**Table 2:** Characteristics of the study population by dental care.

Characteristics	All	Regular dental care	Irregular dental care	*p*
	*n* (%) (column)	*n* (%) (row)	*n* (%) (row)	
All	136 (100.0)	39 (28.7)	97 (71.3)	
Sex				0.173b
Female	54 (39.7)	19 (35.2)	35 (64.8)	
Male	82 (60.3)	20 (24.4)	62 (75.6)	
Education				0.057b
Elementary school	49 (36.8)	11 (22.4)	38 (77.6)	
Upper secondary/Vocational	67 (50.4)	19 (28.4)	48 (71.6)	
Higher education	17 (12.8)	9 (52.9)	8 (47.1)	
Financial situation				0.310b
Good/Average	82 (61.7)	26 (31.7)	56 (68.3)	
Difficult	51 (38.3)	12 (23.5)	39 (76.5)	
Entitled free dental treatment in PDS				**0** **.** **012b**
Yes	72 (52.6)	27 (38.0)	44 (62.0)	
No/Don't know	65 (47.4)	12 (18.5)	53 (81.5)	
Entitlement to reimbursement from the Norwegian National Insurance Scheme				0.436b
Yes	29 (21.2)	10 (34.5)	19 (65.5)	
No/Don't know	108 (78.8)	29 (27.1)	78 (72.9)	
General health				0.160b
Good	57 (41.9)	20 (35.1)	37 (64.9)	
Poor/Average	79 (58.1)	19 (24.1)	60 (75.9)	
Self-reported dental health				**0** **.** **043b**
Good/Average	63 (46.7)	23 (36.5)	40 (63.5)	
Poor	72 (53.3)	15 (20.8)	57 (79.2)	
Substance use				0.286b
Never	44 (32.6)	15 (34.1)	29 (65.9)	
Current/Episodic use	91 (67.4)	23 (25.3)	68 (74.7)	
Smoking				0.067b
Non-Smoker	60 (44.1)	22 (36.7)	38 (63.3)	
Smoker	76 (55.9)	17 (22.4)	59 (77.6)	
Toothbrushing				**0** **.** **014b**
<2/day	64 (47.7)	12 (18.8)	52 (81.3)	
≥2/day	71 (52.6)	27 (38.0)	44 (62.0)	
Xerostomia				0.289b
No/Mild	92 (67.6)	29 (31.5)	63 (68.5)	
Moderate/Severe	44 (32.4)	10 (22.7)	34 (77.3)	
Orofacial pain				0.498b
No pain/Discomfort	62 (45.6)	16 (25.8)	46 (74.2)	
Pain/Discomfort	74 (54.4)	23 (31.1)	51 (68.9)	
MDAS				0.928b
<19	118 (86.8)	34 (28.8)	84 (71.2)	
≥19	18 (13.2)	5 (27.8)	13 (72.2)	
**Age** Median (25-, 75 perc)	35.5 (27.0–47.0)	38.0 (25.0–49.0)	33.0 (27.0–46.5)	0.967a
**Alcohol** Median (25-, 75 perc)	4.5 (1.0–9.0)	2.5 (0.0–6.0)	6.0 (1.0–10.0)	**0** **.** **025a**
**Psychological distress** Median (25-, 75 perc)	2.3 (1.6–2.8)	2.3 (1.7–2.9)	2.2 (1.6–2.7)	0.524a
**PQ-16** Median (25-, 75 perc)	5.0 (3.0–9.0)	6.0 (3.0–9.0)	5.0 (3.0–9.0)	0.723a
**Pain experience at dentist** Median (25-, 75 perc)	3.0 (1.0–5.0)	3.0 (0.0–5.3)	3.0 (1.0–5.0)	0.822a
**Adverse dental experiences without scary stories** Median (25-, 75 perc)	2.0 (1.0–4.0)	2.0 (1.0–4.0)	2.0 (1.0–4.0)	0.347a
**DT** Median (25-, 75 perc)	3.0 (1.0–7.0)	2.0 (0.0–6.0)	3.0 (1.0–8.0)	0.096a
**FT** Median (25-, 75 perc)	7.0 (4.0–11.0)	9.0 (6.0–14.0)	6.0 (4.0–9.5)	**0** **.** **015a**
**MT** Median (25-, 75 perc)	1.0 (0.0–4.0)	0.0 (0.0–3.0)	1.0 (0.0–4.0)	0.445a

Statistically significant *p*-values (*p* < 0.05) are shown in bold. *P*-values were calculated using the Mann–Whitney U test for continuous variables (a) and Pearson's Chi-square test for categorical variables (b).DT, decayed teeth; FT, filled teeth; MT, missing teeth. Totals may vary due to missing data.

Just over half of the participants were entitled to free dental care within the PDS. Irregular dental care was more common among participants without or uncertain about their entitlement, as well as among those reporting poor dental health, toothbrushing less than twice a day, higher alcohol consumption, and fewer filled teeth (Table 2). Supplementary analyses showed that higher AUDIT-C scores were significantly associated with current or episodic use of other substances (OR = 1.21, 95% CI, 1.09–1.34, *p* < 0.001; Supplementary Table S1).

### Univariate regression analysis

Results from the univariate logistic regression analyses are presented in Supplementary Table S2. In univariate analyses, lower education attainment, no, or unknown entitlement to free dental care, poor self-reported dental health, high alcohol use, and low toothbrushing frequency were associated with irregular dental attendance.

### Multivariable block-wise logistic regression

Variables were entered sequentially in conceptual blocks reflecting Andersen's Behavioral Model. The results of the block-wise logistic regression analysis are presented in Table 3.

**Table 3:** Multivariable logistic regression models of irregular dental care with block-wise entry of predictors.

Predictors		OR (95%CI)	*p*-value
**Model 1**	Sex (male)	1.52 (0.69–3.39)	0.299
	Age	1.01 (0.98–1.04)	0.701
*Predisposing factors*	Education		0.081
	Higher education	Reference	
	Upper secondary/Vocational	2.70 (0.86–8.54)	0.090
	**Elementary**	**4.19 (1.19–14.64)**	**0.025**
**Model 2**	Sex (male)	1.55 (0.68–3.51)	0.296
	Age	1.00 (0.97–1.04)	0.821
*Adding enabling factors: No/Unknown entitlement to free dental treatment*	**Education**		**0.045**
	Higher education	Reference	
	Upper secondary/Vocational	2.88 (0.89–9.36)	0.079
	**Elementary**	**5.16 (1.42–18.77)**	**0.013**
	**No/Unknown entitlement to free dental treatment**	**2.91** **(1.25–6.79)**	**0** **.** **014**
**Model 3** **Parsimonious model**	Sex	1.39 (0.58–3.32)	0.458
	Age	1.01 (0.98–1.04)	0.595
*Adding behavioral factors: Alcohol, Smoking, Toothbrushing frequency*	**Education**		0.111
	Higher education	Reference	
	Upper secondary/Vocational	1.92 (0.54–6.91)	0.316
	**Elementary**	**4.13 (1.03–16.62)**	**0.046**
	**No/Unknown entitlement to free dental treatment**	**3.15** **(1.30–7.64)**	**0** **.** **011**
	Alcohol	1.11 (0.99–1.24)	0.069
	Smoking	1.43 (0.57–3.60)	0.449
	**Toothbrushing frequency**	**2.53** **(1.06–6.02)**	**0** **.** **036**
**Model 4** **Extended model**	Sex	1.49 (0.61–3.65)	0.386
	Age	1.01 (0.98–1.04)	0.581
*Adding need factors: Self-reported dental health, DMFT*	Education		0.124
	Higher education	Reference	
	Upper secondary/Vocational	1.87 (0.51–6.86)	0.344
	Elementary	4.00 (0.97–16.40)	0.054
	**No/Unknown entitlement to free dental treatment**	**2.97** **(1.21–7.29)**	**0** **.** **017**
	Alcohol	1.11 (0.99–1.24)	0.074
	Smoking	1.37 (0.54–3.50)	0.509
	Toothbrushing frequency	2.37 (0.98–5.74)	0.056
	Self-reported dental health	1.43 (0.57–3.58)	0.445
	DMFT	1.00 (0.94–1.06)	0.890

Odds ratios (OR) with 95% confidence intervals (CI) are presented. For continuous variables (age, alcohol use, and DMFT), ORs represent the change in odds per one-unit increase. Models were constructed using block-wise entry of predictors: Model 1 included predisposing factors; Model 2 additionally included enabling factors; Model 3 additionally included behavioral factors; and Model 4 additionally included need-related factors. Reference categories are indicated for categorical variables. Statistically significant *p*-values and odds ratios (*p* < 0.05) are shown in bold.

OR, odds ratio; CI, confidence interval; DMFT, decayed, missing, and filled teeth.

Predisposing factors alone (Model 1) did not significantly explain variation in dental attendance. The addition of enabling factors in Model 2 significantly improved the model fit (*Δχ*^2^ = 6.53, df = 1, *p* = 0.011), and the overall model became statistically significant (*χ*^2^ = 13.62, df = 5, *p* = 0.018), explaining 14.2% of the variance (Nagelkerke R^2^ = 0.142).

The inclusion of behavioral factors in Model 3 further improved the model (*Δχ*^2^ = 10.12, df = 3, *p* = 0.018), increasing the explained variance to 23.8% (Nagelkerke R^2^ = 0.238). Model 3 overall was statistically significant (*χ*^2^ = 23.73, df = 8, *p* = 0.003).

Adding need factors in Model 4 did not improve the model fit significantly (*Δχ*^2^ = 0.59, df = 2, *p* = 0.746). Although the final model remained statistically significant (*χ*^2^ = 24.33, df = 10, *p* = 0.007), the increase in explained variance was minimal (Nagelkerke R^2^ = 0.243).

The Hosmer–Lemeshow test indicated acceptable model fit across all models (*p* > 0.05). Based on these findings, Model 3 was retained as the most parsimonious model. Lack of entitlement to free dental care in the PDS, toothbrushing less than twice a day, and elementary school as highest educational attainment remained significantly associated with irregular dental care (Table 3, Model 3).

### Sensitivity analyses

Sensitivity analyses were conducted to examine whether the associations observed in the final model were sensitive to the choice of dental status variable (Supplementary Table S3). In the primary model, dental status was represented by the DMFT index. To assess robustness, DMFT was replaced with its individual components (DT, MT, and FT), while all other variables in the model were kept unchanged.

The estimates for the main predictor, entitlement to free dental treatment, remained stable across models, with odds ratios ranging from 2.97 to 3.17 (0%–6.7% change). Similarly, estimates for alcohol consumption and toothbrushing frequency showed minimal variation (<8%). The estimate for education showed greater variability (−12% to +17.5%). Overall, the results were consistent across model specifications. In addition, bootstrap analyses of the parsimonious and extended models produced similar parameter estimates and did not materially alter the overall interpretation of the findings (Supplementary Table S4).

## Discussion

The current study explored use of and experiences with dental health services among inpatients with psychiatric and/or substance use disorders, and factors associated with regular dental care. Regular dental attendance was uncommon, with more than half seeking dental services only for acute problems or never. Across several measures and in regression models, financial constraints were most clearly associated with low utilization of dental health services. However, dental anxiety was also reported as a barrier to dental care by many, and negative experiences with dental treatment were common.

The financing of dental care in Norway may help explain some of the observed patterns of service use. Most adults pay for dental services out of pocket, but some medical conditions or treatments give rights to free dental care in the PDS or partial reimbursement of costs in public and private dental practices (42–44). The legislation is complex, and entitlements for patients in psychiatric or substance use treatment depend on factors such as duration of institutional stay or receipt of home-based care and require updated documentation. Individuals with substance use disorders may be eligible for free or subsidized care through specific schemes, such as opioid substitution treatment or municipal health services. As a result, rights to care may be fragmented, time-limited, and difficult to navigate. The responsibility for activating and maintaining these entitlements may therefore rest heavily on patients who often have difficulties managing complex administrative systems. This may contribute to uncertainty about entitlements and reduced utilization of dental services. The substantial proportion of participants who reported being uncertain about their entitlement to free dental care may also reflect limited awareness of available benefits. Such uncertainty may itself act as a barrier to accessing care, emphasizing that not only the structural availability of services, but also patients' awareness and understanding of their entitlements, are important for utilization.

This study used Andersen's Behavioral Model of Health Service Use to assess and interpret our findings. Within this model, predisposing factors such as educational attainment had limited explanatory value. In contrast, enabling factors, particularly entitlement to free dental care in the PDS, substantially improved the model and remained significant across all models, highlighting the importance of structural access to care. Notably, half of the respondents had postponed dental visits due to financial constraints in the past two years, highlighting that affordability is a key facilitator of service use. The inclusion of behavioral factors further improved model fit. Toothbrushing frequency was associated with regular dental care, while the association with education was attenuated, suggesting that differences in health-related behaviors may partly underlie observed sociodemographic patterns (45). Alcohol use was associated with irregular dental care in bivariate analyses but not in the final adjusted model. The association between alcohol use and other substance use suggests that alcohol risk may partly reflect broader patterns of polysubstance use rather than an isolated behavioral factor.

Need-related factors showed limited explanatory value in the multivariable models. The study population had substantial oral health issues, including untreated dental caries, oral pain, xerostomia and impaired oral health-related quality of life, as previously reported (2–6, 9, 46). Despite these problems, neither perceived nor clinically assessed dental treatment need was associated with regular dental care, suggesting that even a high accumulation of oral health problems is insufficient to overcome structural barriers to care. Sensitivity analyses replacing DMFT with DT, MT, or FT produced consistent results, suggesting that clinical oral health status alone did not explain whether participants attended dental services regularly. Pain may represent an additional aspect of need not fully captured by these measures. More than 50% of participants reported orofacial pain. However, this did not translate into more regular attendance. This further supports the notion that symptom burden may be insufficient to overcome barriers to care in this population and suggests a mismatch between need and service utilization. The lack of association between clinical need and regular dental care contrasts with findings from the general population, where need is typically a stronger driver of care-seeking behavior (23). In populations with severe psychiatric and substance use disorders, other health- and social challenges may reduce the priority given to oral health, even in the presence of symptoms. In such contexts, structural facilitators, such as entitlement to free dental treatment, may be necessary to translate need into actual service use.

Dental anxiety, estimation of pain related to dental visits, and adverse dental experiences were not statistically associated with regular dental care in this study. This does not mean that psychological barriers were irrelevant. The prevalence of high dental anxiety (MDAS ≥ 19) was 13.2%, which was higher than reported in both a Norwegian population-based study from the Tromsø 7 Study (2.9%) (22), and a UK general population study by Humphris et al. (11.6%) (47). Comparable prevalence estimates (10.6%) have been reported in Finnish urgent dental care patients (48). However, prevalence estimates should be interpreted cautiously, as studies differ with regard to population characteristics, cultural context, scoring approaches, and cut-off values (49).

One third of the participants had postponed dental visits due to fear, and the majority reported previous negative dental experiences, including feelings of fear, helplessness, shame, and discomfort during treatment. These experiences may shape how dental care is perceived, tolerated, and avoided, even if they did not distinguish regular from irregular attenders in the present analysis. The MDAS primarily assesses anticipatory anxiety related to specific dental procedures and treatment situations. In this population, barriers to dental care may also involve shame, helplessness, distrust, loss of control, and broader difficulties engaging with healthcare services. Furthermore, many individuals in this population have limited and irregular contact with dental services, which may reduce the relevance of hypothetical treatment scenarios typically used in dental anxiety measures. Thus, the MDAS may not fully reflect how fear and avoidance are experienced in this population. Taken together, this suggests that psychological and relational barriers may still play an important role but are not captured as independent predictors of regular attendance in this population (50). Thus, although reducing financial barriers may be an important component of improving access, such measures alone are unlikely to fully address the complex and interacting barriers faced by this population. This is further supported by our findings that relational and organizational aspects, such as having a regular dentist, feeling safe during treatment, receiving sufficient information, and experiencing a sense of control were rated as very important by many participants for being able to receive dental care. This may be particularly relevant in the context of prior coercive or involuntary treatment experiences, which could influence trust in healthcare providers (26). Furthermore, about 40% rated dental care under sedation or general anesthesia as important or very important, possibly reflecting the need for additional support in tolerating treatment situations. These findings suggest that access to dental care in this population extends beyond financial coverage alone. Interpersonal and practical support factors may represent important facilitators of care. Although not directly tested as predictors of attendance, these factors provide important contextual insight into how access may be experienced and supported.

## Strength and limitations

This study has several limitations that should be considered. The use of a convenience sample and the absence of information on the number of eligible patients approached or those who declined participation limit the ability to assess potential selection bias and preclude calculation of a response rate. However, this approach was considered necessary given the vulnerable population, where repeated recruitment attempts could be experienced as coercive or distressing.

The modest sample size (*N* = 138) limited statistical power and resulted in some imprecision in the estimates, as reflected by wide confidence intervals. In addition, the cross-sectional design precludes conclusions about causality. Future longitudinal studies are needed to better understand temporal relationships and potential causal pathways between oral health, access to care, and dental service utilization in this population.

Measures of dental attendance and entitlement to care were self-reported and may be subject to misclassification, particularly given the complexity of entitlement systems.

Furthermore, the study was conducted among inpatients in specialist mental health and substance use services, and the findings are therefore not generalizable to outpatients or the general population. The organization of dental care and entitlement systems is also context-specific to Norway, which may limit transferability to other settings.

This study also has important strengths. It was conducted in a hard-to-reach inpatient population with substantial oral health needs, providing rare insight into a clinically and socially vulnerable group. The combination of clinical and radiographic examinations with self-reported data strengthens the validity of the findings. All clinical assessments were performed by a single calibrated examiner, ensuring consistency.

The analyses were guided by Andersen's Behavioral Model of Health Service Use, allowing for a structured examination of predisposing, enabling, and need-related factors. A parsimonious modeling approach was applied to reduce the risk of overfitting, and sensitivity analyses demonstrated that the main findings were robust across different specifications of dental status variables.

## Clinical implications

The findings highlight the importance of maintaining and strengthening public dental care entitlements. However, improving access to care in this population likely requires more than financial coverage alone. Feeling safe during treatment, receiving clear explanations, and having a sense of control were frequently reported as important facilitators. Practical support, including assistance with scheduling and follow-up, may further reduce barriers to attendance. Furthermore, approximately 40% of participants considered dental treatment under sedation or general anesthesia to be important or very important, suggesting that adapted treatment modalities and anxiety-reducing approaches may be important for improving access to care in this population.

## Conclusion

Regular dental attendance was uncommon despite substantial oral disease. Entitlement to free dental treatment and behavioral factors were more strongly associated with attendance than clinical need. The findings of this study suggest that improving dental service utilization in this population requires not only financial coverage, but also clearer pathways into care, support in navigating entitlements, and patient-centered approaches addressing safety, continuity, and control. These findings should be interpreted in light of the study's limitations, including the convenience sampling approach, modest sample size, and cross-sectional design.

## Data Availability

The datasets generated and analyzed during the current study are not publicly available due to restrictions under the EU General Data Protection Regulation (GDPR), which prevents these datasets from being made into open access data. However, access to the de-identified data can be granted by the corresponding author upon reasonable request and is subject to strict adherence to privacy protocols. Interested researchers are encouraged to contact the corresponding author with a detailed proposal of their intended use of the data. All requests will undergo an ethical review to ensure compliance with relevant regulations and ethical standards. Requests to access the datasets should be directed to Kristina Kantola: kristina.kantola@tromsfylke.no.
