# Supplementary material for: Dental service utilization among hospitalized patients with psychiatric and/or substance use disorders: a Norwegian cross-sectional study
Source: Front Oral Health. 2026 Aug 10;7:1904583. doi: 10.3389/froh.2026.1904583 (PMC13500311; doi:10.3389/froh.2026.1904583)
Supplement: Supplementary file 1 [file Supplementaryfile1.docx]

**Supplementary materials**

**Supplementary Table S1. Association between alcohol use and substance use**

| **Variable** | B | **OR (95% CI)** | **p** |
| --- | --- | --- | --- |
| Substance use | 0.186 | **1.21 (1.09 – 1.34)** | **<0.001** |

**Table legend:** Results from a univariate logistic regression analysis with current/episodic substance use as the dependent variable and AUDIT-C score as the predictor. Odds ratios represent the change in odds of substance use associated with a one-unit increase in AUDIT-C score. Statistically significant results (p < 0.05) are shown in bold.

**Supplementary Table S2. Univariate analyses of factors associated with irregular dental care**

| **Variable** | **Irregular dental care**  **OR (95% CI)** | **p** |
| --- | --- | --- |
| **Sex** |  |  |
| Female | Reference |  |
| Male | 1.68 (0.79 – 3.57) | p = 0.175 |
| ***Age*** *(continuous)* | 1.00 (0.97 – 1.03) | p = 0.896 |
| **Education** |  |  |
| Higher education | Reference |  |
| Upper secondary/Vocational | 2.84 (0.96 – 8.46) | p = 0.060 |
| Elementary | **3.89 (1.21** – **12.46)** | **p = 0.022** |
| **Financial situation** |  |  |
| Good/Average | Reference |  |
| Poor | 1.51 (0.68 – 3.35) | p = 0.312 |
| **Entitled free dental treatment in the PDS** |  |  |
| Yes | Reference |  |
| No/Don’t know | **2.71 (1.23** – **5.96)** | **p = 0.013** |
| **Entitlement to reimbursement from the Norwegian National Insurance Scheme** |  |  |
| Yes | Reference |  |
| No/Don’t know | 1.41 (0.59 – 3.40) | p = 0.437 |
| **General health** |  |  |
| Good | Reference |  |
| Average/Poor | 1.71 (0.81 – 3.61) | p = 0.162 |
| **Self-reported dental health** |  |  |
| Good/Average | Reference |  |
| Poor | **2.19 (1.02** – **4.70)** | **p = 0.045** |
| **Psychological distress** *(continuous)* | 0.81 (0.49 – 1.33) | p = 0.400 |
| **PQ-16 score** *(continuous)* | 0.97 (0.89 – 1.07) | p = 0.574 |
| **MDAS** *(continuous)* | 1.05 (0.98 – 1.13) | p = 0.164 |
| **Alcohol** *(continuous)* | **1.12 (1.02** – **1.23)** | **p = 0.023** |
| Low | Reference |  |
| Moderate | 1.22 (0.50 – 2.99) | p = 0.665 |
| High | **3.50 (1.26** – **9.69)** | **p = 0.016** |
| **Substance use** |  |  |
| Never | Reference |  |
| Sometimes/Often | 1.53 (0.70 – 3.34) | p = 0.287 |
| **Smoking** |  |  |
| Never/previous | Reference |  |
| Sometimes/Current | 2.00 (0.95 – 4.27) | p = 0.069 |
| **Toothbrushing frequency** |  |  |
| 2 times a day or more | Reference |  |
| 1 time a day or less | **2.66 (1.21** – **5.86)** | **p = 0.015** |
| **Xerostomia** |  |  |
| No/little | Reference |  |
| Moderate/Severe | 1.57 (0.68 – 3.59) | p = 0.291 |
| **Orofacial pain** |  |  |
| No problems | Reference |  |
| Problems | 0.77 (0.36 – 1.64) | p = 0.499 |
| ***DT*** *(continuous)* | 1.06 (0.97 – 1.16) | p = 0.207 |
| ***FT*** *(continuous)* | **0.92 (0.86** – **0.99)** | **p = 0.022** |
| ***MT*** *(continuous)* | 1.06 (0.97 – 1.16) | p = 0.232 |
| **Adverse dental experiences without scary stories (continuous)** | 1.08 (0.89 – 1.32) | p = 0.436 |
| **Pain experience at dentist** *(continuous)* | 1.01 (0.90 – 1.14) | p = 0.882 |

**Table legend:** Univariate logistic regression analyses examining factors associated with irregular dental care. The outcome variable was dental attendance categorized as less than every second year versus at least every second year. Results are presented as odds ratios (OR) with 95% confidence intervals (CI) and p-values. For continuous variables, ORs represent the change in odds associated with a one-unit increase in the predictor. Statistically significant p-values and odds ratios (p < 0.05) are shown in bold. **Abbreviations:** OR, odds ratio; CI, confidence interval; PDS, Public Dental Service; PQ-16, Prodromal Questionnaire-16; MDAS, Modified Dental Anxiety Scale; DT, decayed teeth; FT, filled teeth; MT, missing teeth.

**Supplementary Table S3. Sensitivity analysis of logistic regression models using DT, MT, and FT**

|  |  | **OR (95%CI)** | **p-value** | **Omnibus** | **Nagelkerke R^2^** | **Hosmer-Lemeshow** |
| --- | --- | --- | --- | --- | --- | --- |
| **Model 4 – DMFT vs DT**  *Add: Need factors, Variable: DT* | Sex | 1.46 (0.60 – 3.55) | 0.407 | Model improvement: Δχ² = 0.72, df = 2, p = 0.699  Total model: χ² = 24.45, df = 10, p = 0.006 | 0.244 (24.4%) | p = 0.454 |
|  | Age | 1.01 (0.98 – 1.04) | 0.590 |  |  |  |
|  | Education  Higher education  Upper secondary/Vocational  Elementary | Reference  1.86 (0.51 – 6.79)  3.91 (0.95 – 16.08) | 0.136  0.347  0.059 |  |  |  |
|  | **No/Unknown entitlement to free dental treatment** | **2.97 (1.21 – 7.28)** | **0.017** |  |  |  |
|  | Alcohol | 1.11 (0.99 – 1.24) | 0.085 |  |  |  |
|  | Smoking | 1.37 (0.54 – 3.47) | 0.513 |  |  |  |
|  | Toothbrushing frequency | 2.33 (0.96 – 5.65) | 0.061 |  |  |  |
|  | Self-reported dental health | 1.34 (0.54 – 3.35) | 0.533 |  |  |  |
|  | DT | 1.02 (0.92 – 1.14) | 0.704 |  |  |  |
| **Model 4 – DMFT vs MT**  *Add: Need factors, Variable: MT* | Sex | 1.35 (0.55 – 3.33) | 0.511 | Model improvement: Δχ² = 1.88, df = 2, p = 0.391  Total model: χ² = 25.62, df = 10, p = 0.004 | 0.255 (25.5%) | p = 0.648 |
|  | Age | 1.01 (0.98 – 1.04) | 0.608 |  |  |  |
|  | Education  Higher education  Upper secondary/Vocational  Elementary | Reference  2.24 (0.60 – 8.39)  4.70 (1.12 – 19.82) | 0.202  0.231  0.035 |  |  |  |
|  | **No/Unknown entitlement to free dental treatment** | **3.17 (1.28 – 7.90)** | **0.013** |  |  |  |
|  | Alcohol | 1.12 (1.00 – 1.26) | 0.052 |  |  |  |
|  | Smoking | 1.28 (0.50 – 3.28) | 0.601 |  |  |  |
|  | Toothbrushing frequency | 2.21 (0.91 – 5.40) | 0.081 |  |  |  |
|  | Self-reported dental health | 1.34 (0.55 – 3.27) | 0.527 |  |  |  |
|  | MT | 1.06 (0.95 – 1.18) | 0.274 |  |  |  |
| **Model 4 – DMFT vs FT**  *Add: Need factors, Variable: FT* | Sex | 1.59 (0.65 – 3.93) | 0.312 | Model Improvement: Δχ² = 3.47, df = 2, p = 0.177  Total model: χ² = 27.21, df = 10, p = 0.002 | 0.269 (26.9%) | p = 0.363 |
|  | Age | 1.02 (0.98 – 1.05) | 0.409 |  |  |  |
|  | Education  Higher education  Upper secondary/Vocational  Elementary | Reference  1.82 (0.49 – 6.85)  3.52 (0.83 – 14.94) | 0.202  0.373  0.088 |  |  |  |
|  | **No/Unknown entitlement to free dental treatment** | **3.03 (1.23 – 7.48)** | **0.016** |  |  |  |
|  | Alcohol | 1.11 (0.98 – 1.24) | 0.093 |  |  |  |
|  | Smoking | 1.41 (0.55 – 3.66) | 0.475 |  |  |  |
|  | Toothbrushing frequency | 2.19 (0.90 – 5.34) | 0.086 |  |  |  |
|  | Self-reported dental health | 1.59 (0.64 – 3.92) | 0.318 |  |  |  |
|  | FT | 0.93 (0.86 – 1.01) | 0.089 |  |  |  |

**Table legend:** Sensitivity analyses were conducted by replacing DMFT with its individual components: decayed teeth (DT), missing teeth (MT), and filled teeth (FT). Odds ratios (OR) with 95% confidence intervals (CI) are presented. Models correspond to the fully adjusted model (Model 4), with each component entered separately as a need factor. Model fit is assessed using the Omnibus test of model coefficients, Nagelkerke R², and the Hosmer–Lemeshow test. Statistically significant p-values and odds ratios (p < 0.05) are shown in bold.
